# GAN-Based LiDAR Translation between Sunny and Adverse Weather for Autonomous Driving and Driving Simulation

**DOI:** 10.3390/s22145287

**Published:** 2022-07-15

**Authors:** Jinho Lee, Daiki Shiotsuka, Toshiaki Nishimori, Kenta Nakao, Shunsuke Kamijo

**Affiliations:** 1 Emerging Design and Informatics Course, Graduate School of Interdisciplinary Information Studies, The University of Tokyo, 4 Chome-6-1 Komaba, Meguro City, Tokyo 153-0041, Japan; shiotsuka@kmj.iis.u-tokyo.ac.jp; 2 Mitsubishi Heavy Industries Machinery Systems Ltd., 1-1, Wadasaki-cho 1-chome, Hyogo-ku, Kobe 652-8585, Japan; toshiaki.nishimori.23@mhi.com; 3 Mitsubishi Heavy Industries Ltd., 1-1, Wadasaki-cho 1-chome, Hyogo-ku, Kobe 652-8585, Japan; kenta.nakao.r2@mhi.com; 4 The Institute of Industrial Science (IIS), The University of Tokyo, 4 Chome-6-1 Komaba, Meguro City, Tokyo 153-0041, Japan

**Keywords:** LiDAR-to-LiDAR translation, adverse weather, autonomous driving, driving simulator, data augmentation, generative adversarial networks

## Abstract

Autonomous driving requires robust and highly accurate perception technologies. Various deep learning algorithms based on only image processing satisfy this requirement, but few such algorithms are based on LiDAR. However, images are only one part of the perceptible sensors in an autonomous driving vehicle; LiDAR is also essential for the recognition of driving environments. The main reason why there exist few deep learning algorithms based on LiDAR is a lack of data. Recent translation technology using generative adversarial networks (GANs) has been proposed to deal with this problem. However, these technologies focus on only image-to-image translation, although a lack of data occurs more often with LiDAR than with images. LiDAR translation technology is required not only for data augmentation, but also for driving simulation, which allows algorithms to practice driving as if they were commanding a real vehicle, before doing so in the real world. In other words, driving simulation is a key technology for evaluating and verifying algorithms which are practically applied to vehicles. In this paper, we propose a GAN-based LiDAR translation algorithm for autonomous driving and driving simulation. It is the first LiDAR translation approach that can deal with various types of weather that are based on an empirical approach. We tested the proposed method on the JARI data set, which was collected under various adverse weather scenarios with diverse precipitation and visible distance settings. The proposed method was also applied to the real-world Spain data set. Our experimental results demonstrate that the proposed method can generate realistic LiDAR data under adverse weather conditions.

## 1. Introduction

For autonomous driving, the most important element is the perception of the surrounding environment through various sensors. Representative perception tasks include object detection [1,2], segmentation [3,4,5] and depth estimation [6,7,8]. For image processing, it was recently shown that deep learning approaches can provide high performance and accuracy in perception tasks. However, the performance of perception tasks using other sensors generally does not follow that obtained when using a camera. The most common reason for this is difficulties in data collection. Most deep learning-based methods require large amounts of data and annotations. However, other sensors (e.g., LiDAR and radar), are relatively expensive and, in order to collect relevant data, a massive amount of human resources and time is required. These reasons have led to a lack of data for these sensors. Furthermore, it is difficult to collect data for all traffic scenes. Traffic scenes are diverse, depending on the traffic volume, location, time of day, weather, and so on. As such, a human cannot collect and annotate data for all relevant situations.

With the advent of generative adversarial networks (GANs) [9], research in the translation field has been expanded in various ways. Translation methods can be mainly divided into two types: supervised and unsupervised methods. Although unsupervised translation methods can achieve powerful performance, their naturalness is less than that of supervised methods. General GAN-based methods focus on image translation; however, LiDAR also provides significant sensor data for autonomous driving. There exist some translation methods considering LiDAR characteristics. However, their main goal is commonly data augmentation, such as translation between simulated LiDAR and real LiDAR [10]. Thus, those methods cannot handle the translation of LiDAR under various weather conditions and are hard to apply to driving simulators.

Driving simulators [11,12,13] have recently been on the rise in the autonomous driving field. They allow people to practice driving as if they were commanding a real vehicle. They have emerged as a potential solution to the need for more data while avoiding the time, cost, and safety issues of current methods. However, the simulated data are unnatural, compared to the actual data, and have insufficient performance for actual driving, especially in terms of LiDAR data. As LiDAR is expensive and the collection of LiDAR data requires a massive amount of human resources and time, the existing LiDAR data are insufficient for learning. Furthermore, LiDAR data are easily influenced by adverse weather, such as rain and fog. However, to the best of our knowledge, no empirical method that can reflect the changes in various weather conditions has been introduced.

**Figure 1:**
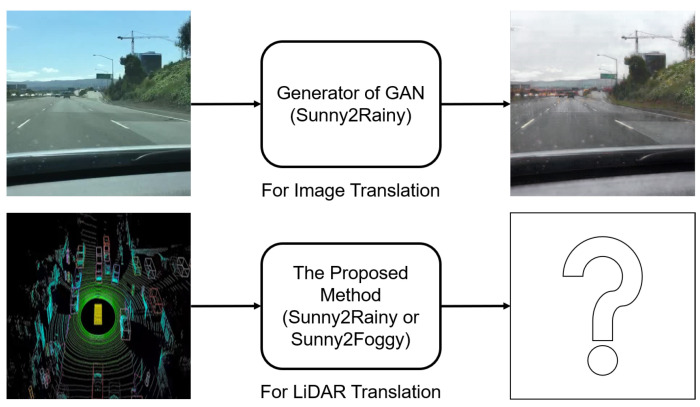
Purpose of the proposed method. As shown in upper figures, GAN-based networks are generally utilized for image translation. Sunny2Rainy indicates the translation from sunny data to rainy data and Sunny2Foggy is that from sunny data to foggy data. We present the first LiDAR-to-LiDAR translation which deals with various adverse weather conditions based on a GAN method.

In this paper, we propose a GAN-based LiDAR translation method that can deal with weather changes. Figure 1 shows the purpose of this study. In Figure 1, we only show the translation from sunny to rainy or foggy. However, we also consider the opposite translation, because there are places where only data with severe weather, such as snow or rain, exist. In those places, the translation from adverse weather to sunny is necessary for data augmentation and verifying algorithms. For the first step, we need to transform LiDAR data as input to the proposed network. To find the optimal data format which gives the best performance, we show the results of various trial-and-error experiments depending on various formats. In addition, we modify the network architecture to handle the LiDAR characteristics, which are different from those of images. The proposed method also uses the JARI data set, which was collected under varying adverse weather conditions, such as precipitation and visible distance. Thus, we can parametrically evaluate the proposed method and demonstrate its performance, in terms of translating sunny to adverse weather.

The contributions of the proposed method are as follows:

- We present the first LiDAR-to-LiDAR translation method based on an empirical method that deals with various adverse weather conditions.
- For the proposed method, we suggest the optimal format for LiDAR translation through several trial-and-error experiments and comparing the results on a case-by-case basis.
- The proposed method deals with translations between sunny and adverse weather, such as rain and fog, with high performance.
- The proposed method is the first LiDAR-to-LiDAR translation which was evaluated parametrically using the JARI data set, which was collected under varying adverse weather conditions, such as precipitation and visible distance.

The remainder of this paper is organized into the following sections. Section 2 introduces the related work in the literature, such as LiDAR translation to handle various weather conditions, LiDAR translation for data augmentation, and that focused on various translation methods. Next, Section 3 presents our proposed method. Then, Section 4 provides the experimental results. Finally, we give our conclusions and discuss directions for future work in Section 5.

## 2. Related Work

### 2.1. LiDAR Translation to Handle Various Weather Conditions

LiDAR is easily affected by adverse weather conditions, such as rain and fog. To determine the influence of weather conditions, many researchers have carried out various studies. In the representative research introduced by Isaac et al. [14] in 2001, the influences of fog and haze on wireless communications were investigated. Furthermore, Rasshofer et al. [15], in 2011, investigated the influence of weather phenomena on automotive LiDAR systems. In recent years, there have been many other works which mentioned the degradation of LiDAR data under different adverse weather conditions [16,17,18,19,20,21,22,23]. Artificial fog simulations are mostly limited to image-based methods. Sakaridis et al. [24] created a foggy version of Cityscapes [25] for semantic segmentation. Furthermore, Hahner et al. [26] leveraged the depth information given in the original data set to improve the performance in the foggy version of the purely synthetic Synscapes [27] data set. Sakaridis et al. also released the ACDC data set [28], which provides semantic pixel-level annotations under adverse conditions. Bijelic et al. [29] recently proposed a first-order approximation for simulating fog in an automotive LiDAR setting. However, their method has a limitation: the simulation only aims to reproduce measurements in a 30 m long fog chamber. The authors in [30,31,32] created LiDAR simulators to deal with adverse weather conditions based on physical models; however, their methods are limited, in that it is impossible to cover all parametric variations in the real world, due to characteristics of the physical models.

### 2.2. LiDAR Translation for Data Augmentation

There exist some generative networks for LiDAR translation for data augmentation in various fields, such as autonomous driving and aerial vehicles [33]. The representative study for LiDAR data augmentation is [10], in which real LiDAR data were generated from simulated LiDAR data by employing CycleGAN [34] to learn the mapping. They considered various LiDAR inputs, such as Bird-eye View (2D BEV) and Polar-Grid Map (2D PGM), to translate between the CARLA [11] and KITTI data sets [35]. However, it cannot formulate the optimal LiDAR input format and also cannot handle weather changes, such as rain and fog. Additionally, there is the LiDAR translation research between LiDAR and radar for data augmentation [36]. PU-GAN [37] learns a rich variety of point distributions from the latent space and up-samples points by constructing an up–down–up expansion unit in the generator. Additionally, a self-attention unit was formulated to enhance feature integration. Its main goal is the enhancement of LiDAR data resolution, such that it also cannot deal with translation between sunny and adverse weather.

### 2.3. Generative Adversarial Networks

The first neural network to generate new data, proposed by Goodfellow et al., was the generative adversarial network (GAN) [9]. Numerous studies have researched derivatives of the GAN. One of the representative generative networks is the WGAN [38], which deals with the data imbalance problem caused by oversampling majority classes. It solves this problem by computing the Wasserstein distance between the distribution of the generated samples and the training data. LSGAN [39] is the first generative network applying MSE loss as the loss function, which improves learning stability. InfoGAN [40] learns interpretable representations by adding latent variables to the input. Conditional GAN (cGAN) [41] explicitly generates the output to be generated by utilizing the label information of the training data. DCGAN [42] utilizes convolutional layers to enhance the expressiveness of the generator and discriminator, in contrast to the original GAN, which was composed of MLPs. SAGAN [43] applies the self-attention mechanism to the structure of the GAN to reflect global features. Pix2Pix [44] utilizes cGAN [41] to learn a mapping from input to output. Similar methods with supervised data for the output have been used for many tasks; however, they cannot deal with the problem of no supervised data and require paired data in a similar environment. CycleGAN [34], DiscoGAN [45], and DualGAN [46] perform domain translation by applying cycle consistency. They do not require paired data. In UNIT [47] and CoGAN [48], two domains are assumed to share a common latent space. Not only these methods, but also several other studies have been proposed to deal with multi-domain translation. Although these methods have achieved powerful performance, most of the generative networks focus only on image translation.

In this work, we present the first LiDAR translation method that can deal with translation between sunny and adverse weather based on an empirical approach, not a physical approach.

## 3. Proposed Method

In this section, we introduce the proposed method. The proposed method is based on CycleGAN [34]. Our final purpose is to generate realistic LiDAR data under various weather conditions. We first determined the most optimal LiDAR data format for training. As the original CycleGAN is intended for image translation, considering the LiDAR data format is a necessary step. There are many elements in LiDAR data. In the proposed method, we utilize LiDAR distance and intensity data for translation. In contrast to the RGB values in a typical image, distance and intensity data of LiDAR are not limited data. Furthermore, the scale of the introduced LiDAR data format is totally different to that of images. To deal with these issues, we designed a network based on the existing CycleGAN structure.

### 3.1. Setup

**Figure 2:**
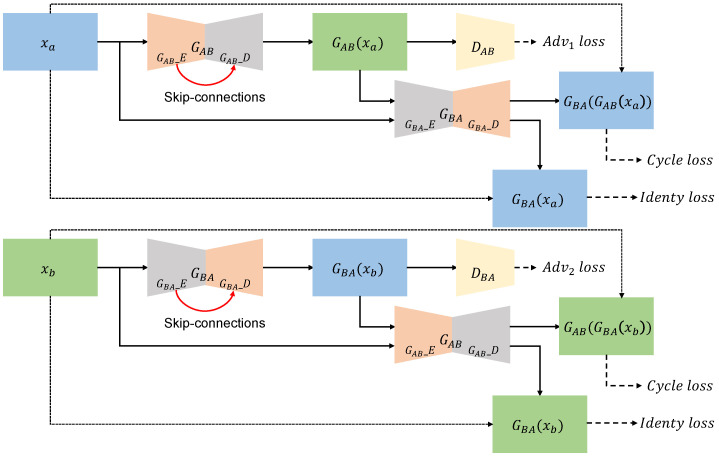
Overview of the proposed method. The blue boxes represent sunny data, and the green boxes represent adverse weather data. $G_{AB}$ is the generator from sunny to adverse weather, and $G_{BA}$ is the generator from adverse weather to sunny. $D_{AB}$ and $D_{BA}$ are the discriminators for each domain. The red arrows below $G_{AB}$ and $G_{BA}$ represent skip connections. The proposed method adopts ResNet-based generators and includes skip connections to improve the performance.

An overview of the proposed model is presented in Figure 2. We denote the real inputs (e.g., sunny, rainy, and foggy) in the source and target domains as $X_{a}$ and $X_{b}$, respectively. Let $G_{AB}$ = $G_{AB}\_E\cdot G_{AB}\_D$ and $G_{BA}$ = $G_{BA}\_E\cdot G_{BA}\_D$ be the source-to-target generator and the target-to-source generator, respectively. In $G_{AB}$ and $G_{BA}$, $G_{AB}\_E$ and $G_{BA}\_E$ are the encoder parts for extracting features from each input, while $G_{AB}\_D$ and $G_{BA}\_D$ indicate the decoders to generate each output. The two discriminators $D_{AB}$ and $D_{BA}$ determine whether a generated image is true or synthetic in the source and target domain, respectively.

### 3.2. Architecture

As the original CycleGAN [34] adopted the ResNet architecture [49], the network has nine residual blocks. While the network with nine residual blocks showed brilliant performance for image translation, it is not suitable for LiDAR translation. Through several experiments, the most optimal architecture for LiDAR translation was found to include a generator with four residual blocks and a discriminator with one residual block. In Section 4, we show the results of several experiments, depending on the number of residual blocks. Additionally, the original CycleGAN adopted the tanh activation function to handle limited RGB values (0–256). As the distance and intensity values in LiDAR data are infinite values, the proposed method utilized the rectified linear unit (ReLU) activation function, instead of the tanh activation function.

The ResNet-based generator in [34] contains two downsizing processes through convolutions. While downsizing has the merit of obtaining global features, it may lose local information. To overcome this problem, the proposed method adopts skip connections [50] in the two downsizing processes. Skip connections can maintain local region information by combining information from lower layers and deeper layers.

### 3.3. Training

Training of the proposed method was highly similar to that of the original CycleGAN [34]. We trained two generators $G_{AB}$, $G_{BA}$ and two discriminators $D_{AB}$, $D_{BA}$ for LiDAR translation between sunny and adverse weather (e.g., rainy or foggy). In the proposed method, the generators $G_{AB}$, $G_{BA}$ and discriminators $D_{AB}$, $D_{BA}$ are trained using the following multiple losses.

In general, a standard GAN [9] contains two networks: a generator and a discriminator. These two networks are trained using the Min-Max game framework. The discriminator distinguishes whether the output, which is produced by the generator, is real or synthetic. In this work, we utilize the standard adversarial loss:

(1) $$\begin{array}{cc} L_{adv_{1}} & =E_{x_{b}∼X_{b}}\left[\log\left(D_{AB}\left(x_{b}\right)\right)\right]\\ & +E_{x_{a}∼X_{a}}\left[\log\left(1-D_{AB}\left(G\left(x_{a}\right)\right)\right)\right], \end{array}$$

(2) $$\begin{array}{cc} L_{adv_{2}} & =E_{x_{a}∼X_{a}}\left[\log\left(D_{BA}\left(x_{a}\right)\right)\right]\\ \; & +E_{x_{b}∼X_{b}}\left[\log\left(1-D_{BA}\left(F\left(x_{b}\right)\right)\right)\right]. \end{array}$$

In unpaired translation, mode collapse is a critical problem, which occurs when a generator cannot generate data samples as diverse as the training data distribution. To deal with this issue, the cycle consistency loss is used, which implicitly enforces diversity. $G_{BA}\left(G_{AB}\left(x_{a}\right)\right)\;\approx \;x_{a}$ and $G_{AB}\left(G_{BA}\left(x_{b}\right)\right)\;\approx \;x_{b}$ are obtained by calculating the $L_{1}$ loss between the original and reconstructed data. This loss is defined as

(3) $$\begin{array}{cc} L_{cyc} & =E_{x_{a}∼X_{a}}\left[|\right|G_{BA}\left(G_{AB}\left(x_{a}\right)\right)-x_{a}{\left|\right|}_{1}]\\ \; & +E_{x_{b}∼X_{b}}\left[|\right|G_{AB}\left(G_{BA}\left(x_{b}\right)\right)-x_{b}{\left|\right|}_{1}]. \end{array}$$

The identity loss is generally utilized to ensure that output is unchanged when data from the target domain are input to the generator:

(4) $$\begin{array}{cc} L_{id} & =E_{x_{a}∼X_{a}}\left[|\right|G_{BA}\left(x_{a}\right)-x_{a}{\left|\right|}_{1}]\\ \; & +E_{x_{b}∼X_{b}}\left[|\right|G_{AB}\left(x_{b}\right)-x_{b}{\left|\right|}_{1}]. \end{array}$$

The final loss is given as follows:

(5) $$L_{total}=L_{adv_{1}}+L_{adv_{2}}+\lambda_{cyc}L_{cyc}+\lambda_{id}L_{id},$$

where $\lambda_{cyc}$ and $\lambda_{id}$ are hyperparameters.

## 4. Experimental Results

**Figure 3:**
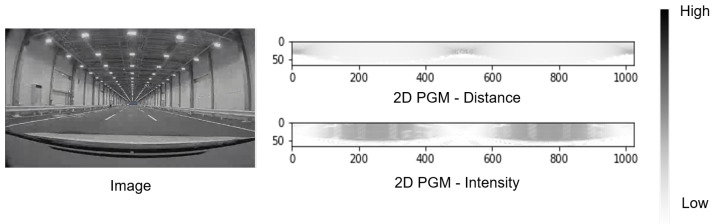
Examples of the JARI driving laboratory and 2D PGM. The left figure shows an example image of the JARI environment where the data are collected. The right ones show the Polar-Grid map (i.e., 2D PGM), which represents the LiDAR 3D point cloud as a 2D grid. The top figure of right ones is an example of 2D PGM for distance and the figure below those is that of 2D PGM for intensity in the JARI environment. Black is higher value and white is lower value in the 2D PGM figures.

For the following experiments, a 2D LiDAR representation was utilized. It is the Polar-Grid map (i.e., 2D PGM), which represents the LiDAR 3D point cloud as a 2D grid, as shown in Figure 3 [10]. The figures show the 2D PGMs for both distance and intensity in the JARI environment where the data are collected. The representation is obtained by encoding both channels and the ray step angle of the LiDAR sensor. The 3D point cloud can be reconstructed from the PGM 2D representation, given enough horizontal angular resolution. There also exists another 2D LiDAR representation, called the Bird-eye View (2D BEV), where the 3D point cloud is projected from above. As the 2D BEV loses height information and the 3D point cloud cannot be reconstructed from it, we do not utilize it in the proposed method.

We experimented with three settings. In the first experiment, we compared the results depending on various LiDAR input formats and architectures, to formulate the most optimal training configuration. Secondly, we experimented with the proposed method depending on skip-connections [50] to show the effectiveness of the proposed method. Finally, we experimented with the proposed method on data collected in the JARI laboratory. The JARI laboratory can control the various weather conditions, such as rainy and foggy, such that we could objectively carry out our experiment.

### 4.1. Data Sets

We mainly utilized the JARI data set, as well as the Spain data set. The JARI data are collected in the JARI driving laboratory, where it can control degrees of various weathers. For the JARI data set, sunny data are divided into dry and wet data, indicating the surface condition of the road. Rain data of the JARI data set are composed of the three settings to deal with precipitation: 30 mm/h, 50 mm/h, and 80 mm/h. Fog data of that also contain three settings to handle the degree of fog: 15 m, 30 m, and 80 m visible distance. These weather data include day and night data, along with two vehicle velocities: 5 km/h and 40 km/h. There are various obstacles in the environments where the data were collected, including real vehicles, real pedestrians, and white lines. Table 1 summarizes the configuration of the JARI data set. The JARI data set was utilized to prove that the proposed method can perform stable translation to adverse weather conditions.

**Figure 4:**
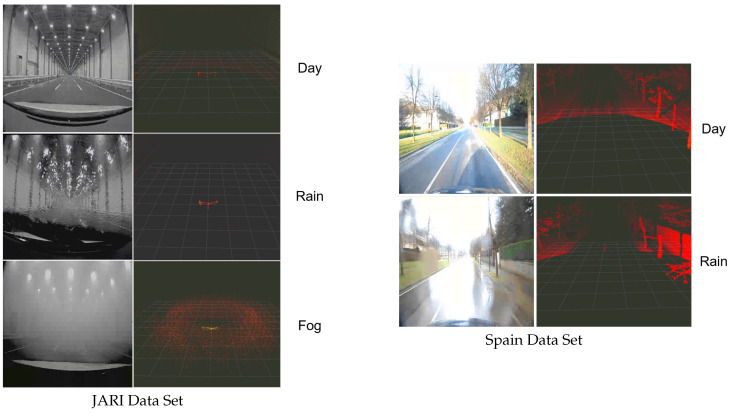
Examples of the JARI and Spain data sets. The JARI data were collected using an Ouster-64, while the Spain data were obtained by an Ouster-128. Thus, the data sets are slightly different. However, we designed the respective architectures separately. Furthermore, fog data do not exist in the Spain data set, due to the climatic characteristics of Spain.

We also utilized the Spain data set to prove that the proposed method can handle various real-world environments. Since the Spain data were collected on real roads in Spain, it cannot control degrees of various weathers. Additionally, due to the climatic characteristics of the country, the Spain data set does not include any fog data. Figure 4 shows examples of the JARI and Spain data sets. The JARI LiDAR data were collected by an Ouster-64 (i.e., Ouster LiDAR with 64 vertical channels), while those in Spain were obtained by an Ouster-128 (i.e., Ouster LiDAR with 128 vertical channels). Thus, the data configurations are slightly different. However, we designed the architectures separately, depending on each LiDAR data set.

### 4.2. Experiment 1: The Most Optimal Input Format and Architecture

For image-to-image translation, inputs consisting of RGB values are common. However, LiDAR data do not contain RGB values and there is no common format for LiDAR training. Thus, we conducted an experiment to determine the most optimal input format and architecture.

LiDAR data are different from image data. While image data contain limited values (e.g., RGB values), LiDAR data consist of unlimited values (e.g., distance, intensity, and reflectivity). In the proposed method, we utilize distance and intensity in LiDAR data, as these values are the most affected by weather and are important for the LiDAR representation. As mentioned above, we utilized the 2D PGM. The input array size was equal to (64,1024,2) for the JARI data set and (128,1024,2) for the Spain data set. The first channel denotes the number of vertical channels, and the second channel indicates the number of horizontal channels. The last channel indicates the number of values, which means distance and intensity values in this study. The distributions of distance and intensity values were 0–250 m and 0–3700, respectively, in the JARI data set.

Table 2 and Table 3 show the results depending on several distances and intensity pairs, with Sunny(dry)–Day and Fog(15 m)–Day in the JARI data set. The result was obtained by summing the errors between the ground truth and the generated result. The equation is given as follows:

(6) $$\begin{array}{c} X_{distance}=\sum_{1}^{all}\left\{\left|D_{G}-D_{T}\right|\right\}, \end{array}$$

(7) $$\begin{array}{c} X_{intensity}=\sum_{1}^{all}\left\{\left|I_{G}-I_{T}\right|\right\}, \end{array}$$

where $X_{distance}$ and $X_{intensity}$ are the sum of distance errors and intensity errors, respectively; $D_{G}$ and $I_{G}$ are distance and intensity values of the ground truth, respectively; and we define $D_{T}$ and $I_{T}$ as the distance and intensity values in the generated result, respectively.

As shown in Table 2 and Table 3, the intensity/15 result outperformed the others. There are two possible reasons for such a result. First, the distance–intensity/15 pair has a distribution highly similar to that of RGB values. As the architecture of the proposed method is based on the CycleGAN [34], which was originally designed for image translation, the results with that pair were superior. Second, when the intensity value is high, the intensity error results (with square root and log) are also likely to increase. Even though the square root and log values are small, the values restored to original intensity could be high, especially when considering high-intensity values.

In the original CycleGAN [34] for image translation, the ResNet block [49] is utilized to extract features in the down-sampling of generator and discriminator. There are nine ResNet blocks in the generator and four ResNet blocks in the discriminator for image translation. As this configuration was designed for image translation, we experimented and compared the results when changing the number of ResNet blocks in each generator and discriminator, as shown in Table 4. We utilized the Sunny(dry)–Day and Fog(80 m)–Day data in the JARI data set. The result was obtained by summing the errors between the ground truth and the generated result, the same as in Table 2 and Table 3. Table 4 indicates that the most optimal architecture for LiDAR translation in the JARI data set was found to be four ResNet blocks for the generator and one ResNet block for the discriminator.

### 4.3. Experiment 2: Advantages of Skip-Connections

In this experiment, we evaluated the effectiveness of skip-connections [50] for LiDAR translation. Skip-connections are connections in deep neural networks that feed the output of a particular layer to later layers in the network. Skip-connections are normally utilized in image training to keep features of lower layers and output higher quality. We expect that these connections allow the proposed method to generate a higher resolution output.

**Figure 5:**
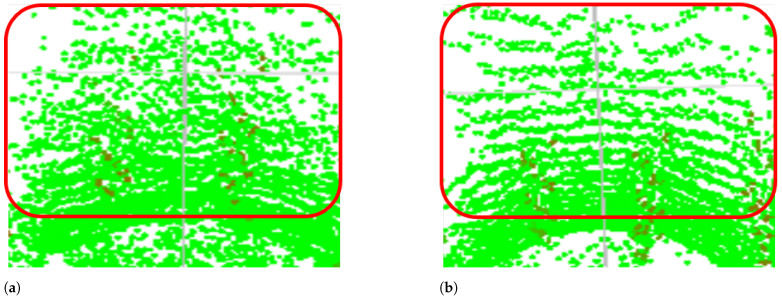
Examples of results without skip-connections (**a**) and with skip-connections (**b**). The results are the reconstructed 3D points seen from above. The green points indicate reconstructed 3D points. The red points indicate the comparatively high value of intensity. While the result without skip-connections did not generate LiDAR rings, that with skip-connections generated LiDAR rings clearly (see the red rectangles for comparison). The red rectangle on the right indicates the LiDAR rings, which are arrangements of LiDAR points that form rings.

We compared the results obtained with and without skip-connections, as shown in Table 5. We utilized the Sunny(dry)–Day and Fog(80 m)–Day data in the JARI data set. The results were obtained by summing the errors between the ground truth and the generated result. The results in Figure 5 are the reconstructed 3D points, as seen above. The green points indicate the reconstructed 3D points. Table 5 and Figure 5 show that the results with skip-connections can generate LiDAR rings clearly, while, without skip-connections, it is hard to represent LiDAR rings (see the red rectangles in Figure 5 for comparison). A LiDAR ring is an arrangement of LiDAR points, which form a ring. Through this experiment, the proposed method with skip-connections outperformed that without skip-connections.

### 4.4. Experiment 3: LiDAR Translation to Handle Adverse Weathers

**Figure 6:**
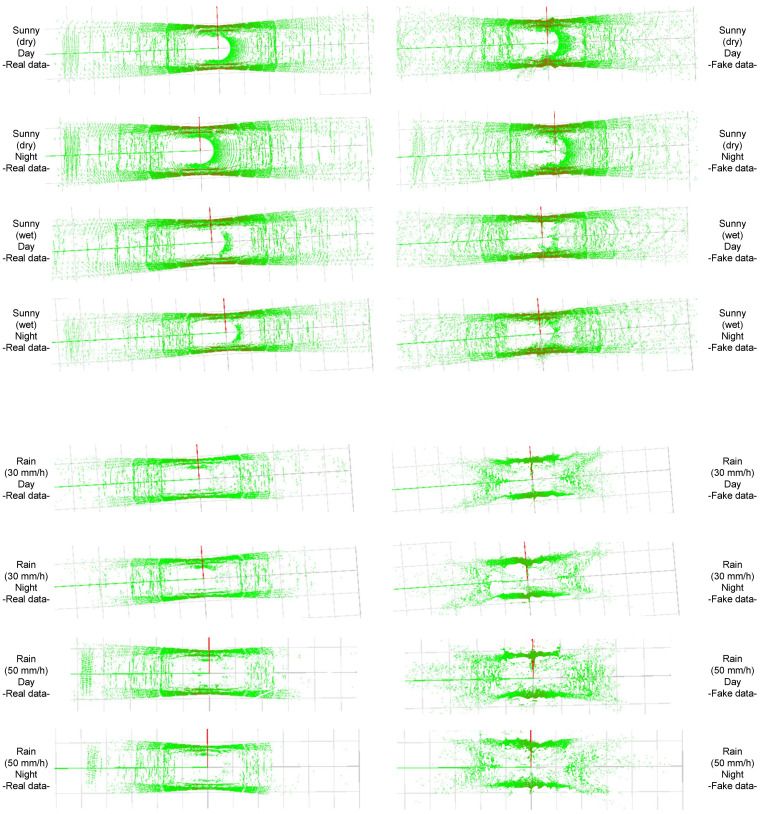
Examples of translation between sunny and adverse weather conditions with the JARI data set. The results are reconstructed 3D points seen from above. The green points indicate reconstructed 3D points. The red points indicate the comparatively high value of intensity. We show the translation results between sunny(wet) and rainy conditions. The images on the left are the real data for the source domains, while those on the right are the generated data for the target domains. Even though there was some noise in the generated data, the proposed method can reflect the changes according to various environmental conditions.

**Figure 7:**
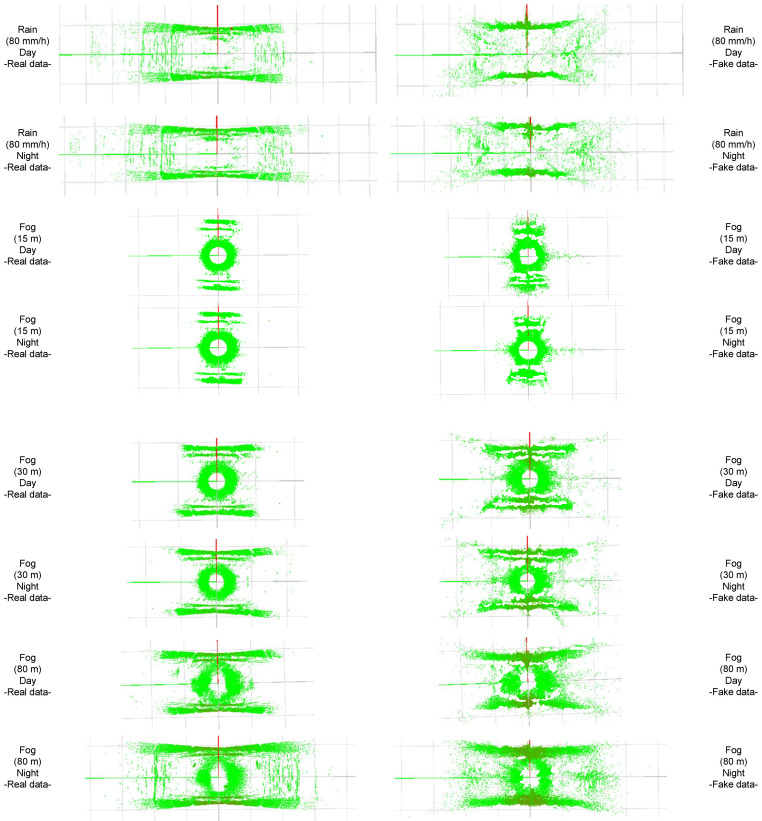
Examples of translation between sunny and adverse weather with the JARI data set. The results are reconstructed 3D points seen from above. The green points indicate reconstructed 3D points. The red points indicate the comparatively high value of intensity. We show the translation results between rainy and foggy conditions. The images on the left are the real data for the source domains, while those on the right are the generated data for the target domains. Even though there was some noise in the generated data, the proposed method can reflect the changes according to various environmental conditions.

In the third experiment, we tested the ability of the proposed method to translate between sunny and adverse weather conditions utilizing the JARI and Spain data sets. The experiment with the JARI data set was carried out for parametrical evaluation, depending on the various degrees of adverse weather conditions. Figure 6 and Figure 7 show example results of translation between sunny and adverse weather conditions using the JARI data set. These results are reconstructed 3D points, as seen above. The green points are the reconstructed 3D points. The images on the left show the real data for the source domains, while those on the right are the generated data for the target domains. Table 6 provides the distance and intensity error results for translation. The results were obtained by summing the errors between the ground truth and the generated result, similarly to the other experiments.

**Figure 8:**
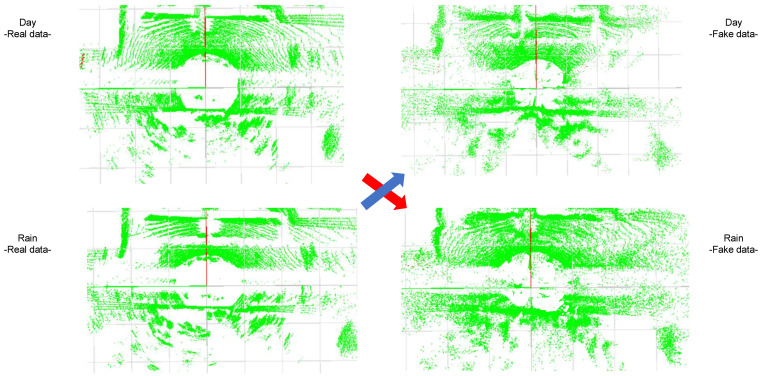
Example of translation between sunny and rainy conditions with the Spain data set. The results are reconstructed 3D points seen from above. The green points indicate reconstructed 3D points. In this figure, we show the translation from sunny to rainy condition. The images on the left are the real data for the source domains, while those on the right are the generated data for the target domains. The synthetic rain data were obtained by translation from the real day data, denoted by the red arrow. In addition, the synthetic day data were generated by translation from real rain data, as shown by the blue arrow. As there is no large difference between the day and rain data, the translation results showed no noticeable difference. We thus confirmed that the proposed method is applicable to real-world scenarios.

The experiment with the Spain data set was carried out to obtain evidence that the proposed method applies to real-world scenarios. Figure 8 shows an example of results using the Spain data set. These results are also reconstructed 3D points, as seen above. As in Figure 6 and Figure 7, the reconstructed 3D points are represented by green points. The images on the left are the real data for the source domains, while those on the right are the generated data for the target domains. The result for the synthetic rain data was obtained by translation from the real day data, as denoted by the red arrow. In addition, the synthetic day data were generated by translation from the real rain data, denoted by the blue arrow. As the Spain data set does not include fog data, due to weather conditions of the country, we only considered translation between sunny and rainy conditions.

As shown in Figure 6, Figure 7 and Figure 8, the proposed method can translate successfully between sunny and adverse weather conditions. By comparing the real data of day and night for all weathers (e.g., the real data pairs of Sunny(dry)/Day–Sunny(dry)/Night and Fog(15 m)/Day–Fog(15 m)/Night) in Figure 6 and Figure 7, we can confirm that the LiDAR data between day and night are nearly the same. Although the timestamps of the real day and night data are slightly different, the arrangement and intensity values of the LiDAR points are almost identical. Through this confirmation, we can also prove that LiDAR is almost not influenced by darkness, according to the basic characteristic of LiDAR. The proposed method can easily translate from the day(dry) to day(wet), as there is no large difference between day(dry) and day(wet). However, the proposed method generated some wrong points when translating between sunny and rainy. This phenomenon also occurred when translating between sunny and foggy. This is because the proposed method may determine high errors in distance and intensity when the difference between the source and target domain is extremely large, such as when translating between rainy and foggy. More wrong points are generated farther away from the position of the LiDAR as the distance and intensity errors are high at those positions, particularly in the translation between rainy and foggy. This is the key limitation of the proposed method.

The proposed method still reflects the changes in the phenomena according to various weather conditions. In particular, the proposed method can successfully express the LiDAR rings, which take the form of a donut shape, in the translation between sunny and foggy conditions. These rings occur when a vehicle passes through fog, as shown in Figure 7. In addition, when it rains, distant points are erased, due to the visible distance of LiDAR. The proposed method also represents this phenomenon clearly when translating from sunny to rainy. Finally, we also showed that the proposed method can deal with real-world environments through the example results provided in Figure 8. As there was no large difference between the day and rain data, the translation results showed no noticeable difference. We further confirmed that the proposed method is applicable to real-world scenarios.

### 4.5. Discussion

In this section, we provide some qualitative assessments of the proposed method. In the first experiment, we compared the translation results of the proposed method, depending on various LiDAR formats and architectures. Through several trials, we confirmed that the proposed method performed the best when the distance and intensity values were similar to RGB values during training. This is also suitable for training, under the condition that the number of ResNet blocks is four for the generator and one for the discriminator. In experiment 2, we showed the effectiveness of skip connections in the proposed model. We showed that the proposed model can prevent the loss of local information in the encoder–decoder model generator. The results show that the proposed method is effective for preserving small regions, such as LiDAR rings. In the third experiment, we showed that the proposed method can perform a successful translation between sunny and adverse weather conditions. In particular, the proposed method successfully represented the LiDAR ring phenomenon, where the LiDAR points present a donut shape, which occurs while passing through the fog, in the translation to foggy conditions. The proposed method can also handle the phenomenon in which distant points are erased under rainy conditions. On the other hand, one limitation of this method is that the distance and intensity errors are relatively high when the gap between source and target domains is extremely big, such as when translating between rainy and foggy. Thus, we will consider this problem in future work. Since rain data in the JARI dataset are slightly different with those in real roads, we will also collect a large amount of rain data variously in real roads and modify the proposed method to optimize.

## 5. Conclusions

We proposed a method to perform LiDAR-to-LiDAR translation to handle adverse weather conditions. Traditional methods for LiDAR translation focus only on translation from simulated to real data. However, the proposed method can deal with translation between sunny and adverse weather data (i.e., rain and fog). Furthermore, the proposed method is very effective, in terms of data augmentation. Some traditional methods for LiDAR translation which can handle various weather conditions have been introduced recently; however, these methods cannot generate natural LiDAR data, as they are based on mathematical formulas. In contrast, the proposed method generates LiDAR data which can represent changes in weather conditions, by training various weather characteristics from the JARI and Spain data sets.

To train LiDAR data with considering various weather characteristics, we formulated the most optimal LiDAR input format and network architecture through diverse experiments. Since traditional translation networks are intended for image translation, formulating the LiDAR data format is a necessary step. Through various comparison experiments by changing LiDAR formats which are composed of distance and intensity values, we figured out that the most optimal format is when it is similar to the distribution of RGB values, while the RGB values in a typical image are limited, distance and intensity values of LiDAR are infinite. To deal with this issue, we designed the architecture with activation functions, which can handle unlimited value. Furthermore, the scale of the proposed LiDAR data format is totally different to that of images. To handle this problem, we revised a network based on the existing CycleGAN structure by modulating ResNet blocks which are utilized for feature extraction.

In our experiments, we used two data sets: JARI and Spain. We observed that the proposed model could generate natural LiDAR data for both environments. We expect that the proposed model can be adopted in driving simulators, for the examination of various autonomous driving algorithms before real-world testing. Thanks to the proposed method, we expect that researchers in the field of autonomous driving can save both time and costs.

In future work, we will improve the proposed method to handle more adverse conditions, such as heavy rain, heavy snow, and heavy fog. As we only tested the proposed method using the real roads in the Spain data set, which includes small weather variations, we, therefore, plan to collect various data from real roads and modify the proposed method to optimize it to real road conditions. 

## Figures and Tables

**Table 1:** JARI data set configuration. “O” indicates that data exist. Rain data are composed of the three settings to deal with precipitation (30 mm/h, 50 mm/h, and 80 mm/h) and fog data also contain three settings to handle the degree of fog (15 m, 30 m, and 80 m visible distance).

Conditions	Day/Night	5 km/h	40 km/h
Sunny (dry)	Day	O	O
	Night	O	O
Sunny (wet)	Day	O	O
	Night	O	O
Rain (30 mm/h)	Day	O	O
	Night	O	O
Rain (50 mm/h)	Day	O	O
	Night	O	O
Rain (80 mm/h)	Day	O	O
	Night	O	O
Fog (15 m)	Day	O	O
	Night	O	O
Fog (30 m)	Day	O	O
	Night	O	O
Fog (80 m)	Day	O	O
	Night	O	O

**Table 2:** Distance error results depending on LiDAR data format. The numbers in the table indicate distance errors (in m). The bold numbers show that the most optimal format is Intensity/15.

From Source to Target	Original	Intensity/15	Intensity Square-Root	$\log_{2}$(Intensity)
from Sunny(dry)–Day to Fog(15 m)–Day	3.29772	**1.89300**	2.00392	2.29613
from Fog(15 m)–Day to Sunny(dry)–Day	11.29231	**5.31886**	6.69213	7.00921

**Table 3:** Intensity error results depending on LiDAR data format. The numbers in the table indicate intensity errors. The bold numbers prove that the most optimal format is Intensity/15.

From Source to Target	Original	Intensity/15	Intensity Square-Root	$\log_{2}$(Intensity)
from Sunny(dry)–Day to Fog(15 m)–Day	41.22198	**18.17874**	22.55924	21.61834
from Fog(15 m)–Day to Sunny(dry)–Day	360.95213	**178.30045**	189.13991	190.81837

**Table 4:** Error results depending on the number of ResNet blocks in the generator and discriminator. G_Num and D_Num indicate the number of ResNet blocks in the generator and discriminator, respectively. The numbers in the table indicate the distance and intensity errors. The bold numbers show that the most architecture is four ResNet blocks for the generator and one ResNet block for the discriminator.

G_Num & D_Num	-Distance Error [m]- from (Fog(80 m)–Day to Sunny(dry)–Day)	-Distance Error [m]- from (Sunny(dry)–Day to Fog(80 m)–Day)	-Intensity Error- from (Fog(80 m)–Day to Sunny(dry)–Day)	-Intensity Error- from (Sunny(dry)–Day to Fog(80 m)–Day)
1_1	4.67128	2.62430	141.66245	97.37588
2_1	3.99260	2.50344	133.39045	90.80555
3_1	4.08508	2.45496	126.61005	87.46522
**4_1**	**3.71519**	**2.40176**	**118.97728**	**87.32463**
4_2	7.30083	2.59537	138.42924	88.39707
5_1	4.35619	2.41132	139.32719	88.23212
6_1	4.60828	2.43462	165.04027	89.37218

**Table 5:** Error results with and without skip-connections. The numbers in the table indicate the distance and intensity errors. The bold numbers prove that the proposed method with skip-connections outperforms that without.

	-Distance Error [m]- from (Fog(80 m)–Day to Sunny(dry)–Day)	-Distance Error [m]- from (Sunny(dry)–Day to Fog(80 m)–Day)	-Intensity Error- from (Fog(80 m)–Day to Sunny(dry)–Day)	-Intensity Error- from (Sunny(dry)–Day to Fog(80 m)–Day)
w/o skip connections	3.71519	2.40176	118.97728	87.32463
**w skip** **connections**	**3.62987**	**2.40018**	**109.62120**	**87.00812**

**Table 6:** Translation results under various conditions with the JARI data set.

From Source to Target	Distance Error [m]	Intensity Error
from Sunny(dry)–Day to Sunny(dry)–Night	1.49497	85.98490
from Sunny(dry)–Night to Sunny(dry)–Day	1.43653	94.33114
from Sunny(dry)–Day to Sunny(wet)–Day	1.75741	85.57328
from Sunny(wet)–Day to Sunny(dry)–Day	1.81523	92.17662
from Sunny(dry)–Day to Sunny(wet)–Night	1.76100	87.66818
from Sunny(wet)–Night to Sunny(dry)–Day	1.84540	94.66367
from Sunny(dry)–Day to Rain(30 mm/h)–Day	2.78518	68.59065
from Rain(30 mm/h)–Day to Sunny(dry)–Day	3.08751	99.64109
from Sunny(dry)–Day to Rain(30 mm/h)–Night	2.74591	65.87693
from Rain(30 mm/h)–Night to Sunny(dry)–Day	3.36074	109.62120
from Sunny(dry)–Day to Rain(50 mm/h)–Day	2.77008	70.83799
from Rain(50 mm/h)–Day to Sunny(dry)–Day	3.47687	112.60500
from Sunny(dry)–Day to Rain(50 mm/h)–Night	2.89603	69.15411
from Rain(50 mm/h)–Night to Sunny(dry)–Day	3.28403	103.78098
from Sunny(dry)–Day to Rain(80 mm/h)–Day	2.69017	59.15411
from Rain(80 mm/h)–Day to Sunny(dry)–Day	3.60374	98.61141
from Sunny(dry)–Day to Rain(80 mm/h)–Night	2.60951	62.18444
from Rain(80 mm/h)–Night to Sunny(dry)–Day	3.15333	96.81059
from Sunny(dry)–Day to Fog(15 m)–Day	1.73134	17.84077
from Fog(15 m)–Day to Sunny(dry)–Day	4.19671	111.54227
from Sunny(dry)–Day to Fog(15 m)–Night	1.69258	16.66186
from Fog(15 m)–Night to Sunny(dry)–Day	4.19542	115.00208
from Sunny(dry)–Day to Fog(30 m)–Day	1.88871	56.95320
from Fog(30 m)–Day to Sunny(dry)–Day	3.62490	109.74681
from Sunny(dry)–Day to Fog(30 m)–Night	1.90341	55.06957
from Fog(30 m)–Night to Sunny(dry)–Day	3.39191	97.75404
from Sunny(dry)–Day to Fog(80 m)–Day	2.20018	67.00812
from Fog(80 m)–Day to Sunny(dry)–Day	2.62987	89.62120
from Sunny(dry)–Day to Fog(80 m)–Night	2.35044	63.26001
from Fog(80 m)–Night to Sunny(dry)–Day	2.57701	85.65783

## Data Availability

The data are available upon request due to restrictions. The data presented in this study are available from the corresponding authors upon request. The data are not publicly available due to the project’s contract.
